# Combining Biomarkers with EMR Data to Identify Patients in Different Phases of Sepsis

**DOI:** 10.1038/s41598-017-09766-1

**Published:** 2017-09-07

**Authors:** Ishan Taneja, Bobby Reddy, Gregory Damhorst, Sihai Dave Zhao, Umer Hassan, Zachary Price, Tor Jensen, Tanmay Ghonge, Manish Patel, Samuel Wachspress, Jackson Winter, Michael Rappleye, Gillian Smith, Ryan Healey, Muhammad Ajmal, Muhammad Khan, Jay Patel, Harsh Rawal, Raiya Sarwar, Sumeet Soni, Syed Anwaruddin, Benjamin Davis, James Kumar, Karen White, Rashid Bashir, Ruoqing Zhu

**Affiliations:** 1 Department of Bioengineering, University of Illinois at Urbana Champaign, 1270 Digital Computer Laboratory, 1304W, Springfield Ave., 61801 Urbana, IL USA; 2 Department of Statistics, University of Illinois at Urbana Champaign, Illini Hall, 725S Wright St #101, 61820 Champaign, IL USA; 3 Biomedical Research Center, Carle Foundation Hospital, 61801 Urbana, IL USA; 4 Prenosis Inc., 210 Hazelwood Drive, Suite 103, 61822 Champaign, IL USA

**Keywords:** Biotechnology, Machine learning, Infectious diseases, Biomarkers, Translational research

## Abstract

Sepsis is a leading cause of death and is the most expensive condition to treat in U.S. hospitals. Despite targeted efforts to automate earlier detection of sepsis, current techniques rely exclusively on using either standard clinical data or novel biomarker measurements. In this study, we apply machine learning techniques to assess the predictive power of combining multiple biomarker measurements from a single blood sample with electronic medical record data (EMR) for the identification of patients in the early to peak phase of sepsis in a large community hospital setting. Combining biomarkers and EMR data achieved an area under the receiver operating characteristic (ROC) curve (AUC) of 0.81, while EMR data alone achieved an AUC of 0.75. Furthermore, a single measurement of six biomarkers (IL-6, nCD64, IL-1ra, PCT, MCP1, and G-CSF) yielded the same predictive power as collecting an additional 16 hours of EMR data(AUC of 0.80), suggesting that the biomarkers may be useful for identifying these patients earlier. Ultimately, supervised learning using a subset of biomarker and EMR data as features may be capable of identifying patients in the early to peak phase of sepsis in a diverse population and may provide a tool for more timely identification and intervention.

## Introduction

Sepsis is a poorly-understood clinical syndrome characterized by dysregulation of the immune system’s response to infection. Precise definitions – as well as reports of mortality rate – have varied widely, yet there is strong consensus that sepsis is one of the most significant contributors to morbidity and mortality worldwide^1–3^. It is the leading cause of death and is the most expensive condition treated in U.S. hospitals, exerting a $20.3 billion burden in 2011, 5.2% of total costs to the healthcare system nationwide^4^.

It has been believed for some time that timely recognition and administration of appropriate treatment (e.g. “early goal-directed therapy”) will ultimately improve outcomes in septic patients^2, 5, 6^. Recent data has characterized the leading causes of sepsis as infections of the respiratory tract, urinary tract, gastrointestinal system, and skin^7^. These data support the notion that a major roadblock to addressing the sepsis syndrome lies in the identification of the septic patient among the most routine of patient presentations. Meanwhile, a more recent study failing to find benefit to early goal-directed therapy highlights the need for more specific tools in the sepsis triage process^8^.

It is clear that a more objective tool for identification of sepsis is desperately needed, both to aid early recognition of the sepsis syndrome as well as to provide clarity to its complex progression and each individual’s response to intervention. Most efforts towards this goal have followed one of two themes:^1^ techniques utilizing the data available in electronic health records for early identification of sepsis using parameters already measured clinically, and^2^ techniques utilizing novel biomarker measurements that are not currently measured clinically. Methods from the first category have varied in sophistication from relatively simple Best Practice Alert (BPA) systems that automatically notify practitioners based on modified SIRS criteria^9–11^ to more sophisticated machine learning algorithms that incorporate hundreds of features in predictive analytics models^12–15^. Most efforts in the latter category have focused on single biomarker studies from critical care patient populations that have yielded mixed results^16–20^. Pierrakos and Vincent^2010^ analyzed 3370 clinical studies that spanned 178 biomarkers and concluded that there is no single biomarker that is able to clearly differentiate sepsis syndrome from an inflammatory response due to other causes^16^. In the past decade, studies have demonstrated the added value of combining multiple biomarkers simultaneously^21–26^.

Because it has long been thought that a complicated interplay of pro and anti-inflammatory biomarkers undergirds the immune dysregulation in sepsis, the natural hypothesis is that measurement of key contributory biomarkers, in addition to traditional clinical measures, may be aggregated to characterize the course of sepsis and provide individualized insight into intervention strategy. This is effectively a combination of the two themes observed in previous work in this area. While a few efforts have attempted to take this approach and demonstrated the added value in combining markers, extensive research remains to be done in investigating the interplay of biomarkers and clinical measurements on a large and diverse patient population^27, 28^

**Figure 1:**
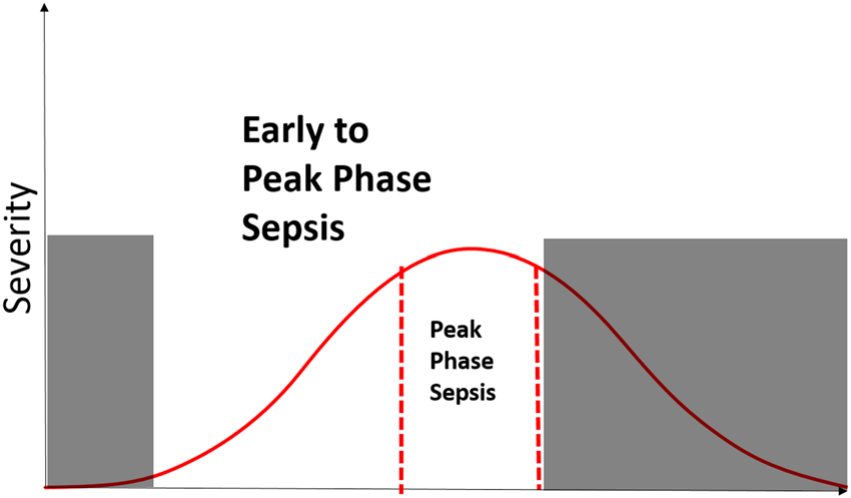
Early to Peak Phase Sepsis Illustration. The red curve represents a hypothetical patient’s severity of sepsis as a function of time, where the apex of the curve corresponds to their worst case state. A patient is considered to be in the early to peak phase of sepsis if they exist on a point on the curve that is not shaded. We marked the boundaries of peak phase sepsis to illustrate the fact that a patient is in the peak phase of sepsis if they are within some tolerance of their worst case state.

The literature has also maintained the notion that sepsis is characterized by a dysregulation of the immune system which fluctuates in severity over time. However, most clinical studies overlook the temporal aspect of sepsis and simply label their patients as being septic or non-septic, regardless of the patient’s stage in the course of the disease. In most clinical use scenarios, the primary concern is to rapidly identify patients that are deteriorating in condition. With this in mind, we have developed a model to identify patients who are traversing from a relatively healthy state to within their most severe septic state (Fig. 1). We refer to this critical period as the “early to peak phase” of sepsis. In all cases, the state of the patient was determined by retrospective clinical adjudication. We believe such a model is more powerful and clinically relevant as it not only discriminates patients in the early to peak phase of sepsis from non-septic patients but also from patients in the recovery phase of sepsis.

In addition to predicting if a patient is in the early to peak of sepsis, we defined further subdivided phases of sepsis and analyzed how our model performed at finer granularities of categorization and which features were most relevant in predicting these categories. Further information behind the adjudication process and categories are described in Materials and Methods.

Since we hypothesize that biomarker measurements combined with electronic medical record data may provide insight into sepsis and its progression, we applied various machine learning (ML) algorithms using multiple biomarker measurements from a single blood sample and EMR data to predict whether or not a patient is in the early to peak phase of sepsis. Algorithms trained on these data provide evidence that biomarkers do provide concrete predictive power and using a subset of biomarkers and EMR data as features may be capable of earlier identification of deteriorating septic patients in a diverse population. Furthermore, by categorizing sepsis into finer granularities, we were able to evaluate model performance as a function of category and analyze which biomarkers and subsets of EMR data best discriminated a given category from others. This ultimately provides scientific insights regarding the immune response as a function of all individual categories.

## Results

### Patient population

Venous blood samples were collected from 444 hospitalized adult (age ≥18) patients at Carle Foundation Hospital for whom hospital providers had ordered blood cultures over the course of May 2014 to May 2016. Characteristics of this patient population obtained from the EMR are summarized in Table 1.

**Table 1:** Patient characteristics where septic population is determined by clinical adjudication labels. Blood culture results and SIRS criteria measurements reported were taken at the time of the biomarker measurement.

Characteristic		Early to Peak Phase Sepsis Population (N = 76)	Non-septic and Recovery Phase SepsisPopulation (N = 368)
**Age, SD**		61, 19	61, 20
**Gender**		47% male, 53% female	56% male, 44% female
**Chronic Conditions**	Cancer	20%	17%
	Diabetes	29%	19%
	Chronic Kidney Disease	9%	9%
	COPD	12%	10%
	Asthma	1%	1%
**Infection (i.e pneumonia, bronchitis, meningitis)**		89%	67%
**Most common infections**	Pneumonia	28%	23%
	Cellulitis	14%	19%
	UTI	41%	19%
	GI infections	11%	5%
**Positive Blood Culture**		38%	22%
**SIRS (at least 2/4 criteria)**		93%	64%
	SIRS (exactly 2/4 criteria)	25%	33%
	SIRS (exactly 3/4 criteria)	39%	25%
	SIRS (exactly 4/4 criteria)	29%	8%
**Infection** **+** **SIRS (at least 2/4 criteria)**		79%	42%
	Infection + SIRS (exactly 2/4 criteria)	22%	22%
	Infection + SIRS (exactly 3/4 criteria)	36%	16%
	Infection + SIRS (exactly 4/4 criteria)	21%	4%
**SOFA score, SD**		3.77, 3.39	2.17, 2.45
**qSOFA score, SD**		1.46, .92	1.07, .94

### Predictive power of individual biomarkers

Based on the paradigm that sepsis can be biochemically described as an interplay of pro and anti-inflammatory processes, we chose a collection of biomarkers based on the current literature^28, 29^ that were expected to be implicated in these processes and thus variably elevated in blood plasma during the course of the disease. The following biomarkers were measured: tumor necrosis factor α (TNF-α), interleukins 1β, 6, 1ra, and 18 (IL-1β, IL-6, IL-1ra IL-18), granulocyte colony-stimulating factor (G-CSF), procalcitonin (PCT), serum triggering receptor expressed on myeloid cells-1 (sTREM-1), matrix metallopeptidase 9 (MMP-9), tumor necrosis factor receptors 1 and 2 (TNFR1, TNFR2), interferon-γ-inducible protein 10 (IP-10), monocyte chemoattractant protein 1 (MCP-1), neutrophil gelatinase-associated lipocalin (NGAL), and neutrophil cluster of differentiated 64 (nCD64). Concentration of soluble biomarkers was measured in blood plasma and expression level of cell surface markers was measured by flow cytometry.

To first characterize the predictive power of individual biomarkers, we calculated the AUC using each biomarker as an independent early to peak phase sepsis diagnostic marker. These values are listed in Table 2. We also report the AUC determined from individual numeric EMR parameters which are commonly used in the inpatient setting to guide clinical suspicion for sepsis in Table 3.

**Table 2:** AUCs using individual biomarkers as the sole feature. SVMs were used with the clinical adjudication label set to calculate the AUC for each biomarker.

Individual Biomarker	AUC
TNF-α	0.60
IL-1β	0.49
GCSF	0.51
IL6	0.77
PCT	0.71
sTREM1	0.43
IL18	0.44
MMP9	0.42
TNFR1	0.63
TNFR2	0.65
IP10	0.52
MCP1	0.60
IL1ra	0.68
NGAL	0.58
CD64	0.65

**Table 3:** AUCs using individual EMR parameters as the sole feature. SVMs were used with the clinical adjudication label set to calculate the AUC for EMR parameters commonly used in clinical suspicion of sepsis.

Individual EMR Parameters	AUC
Leukocyte Count	0.67
Lactic Acid	0.43
Systolic Blood Pressure	0.43
Pulse	0.54
Temperature	0.45
Respirations	0.40

### Machine learning techniques used on biomarker and EMR data

We trained prediction models on 3 different sets of features: biomarkers alone, EMR data alone, and biomarkers combined with EMR data. We tried 5 different machine learning algorithms (logistic regression, support vector machines (SVM), random forests, adaboost, and naive Bayes) and estimated their prediction performances (AUCs) using repeated 10-fold cross-validation^30^. For each set of features, we repeated this entire procedure 1000 times in order to generate different cross-validation folds. We reported the average cross-validation AUC estimates for each algorithm and each set of features using clinical adjudication labels as the gold standard definition of sepsis (treating patients in categories 2–5 as positive examples and patients in categories 1, 6–11 as negative examples) in Table 4. The first column corresponds to using all 31 features in our model, which consists of 15 non-traditional biomarkers and 16 parameters from the EMR, the second column corresponds to using only the EMR data, and the third column corresponds to using only the non-traditional biomarkers. For each algorithm, we performed feature selection on each of the three set of features and then trained and evaluated our model. For more details behind these feature selection criteria, consult Materials and Methods and the Supplementary Information section. The feature rankings outputted by LASSO, SVM, random forests, and adaboost^31, 32^ are displayed in Table S1. We also report the statistical difference between each algorithm and each feature set relative to our final model in Table S3. Consult Materials and Methods for more information behind the training and comparison of models.

**Table 4:** Algorithm’s AUC as a function of feature set for clinical adjudication labels. The AUC is calculated for each algorithm with and without its respective feature selection method. The EMR data that is used is constrained to up to 48 hours before the biomarker measurement and 1 hour after.

Algorithm	All features	EMR	Biomarkers
Logistic Regression	0.78	0.72	0.78
Logistic Regression w/ feature selection	0.79	0.73	0.79
SVM	0.79	0.73	0.78
SVM w/ feature selection	0.81	0.75	0.80
Random Forest	0.77	0.70	0.74
Random Forest w/ feature selection	0.77	0.72	0.77
Adaboost	0.81	0.75	0.79
Adaboost w/ feature selection	0.81	0.76	0.80
Naive Bayes	0.76	0.69	0.77
Naive Bayes w/feature selection	0.80	0.73	0.79

### Prediction of early to peak phase sepsis with SVM

**Figure 2:**
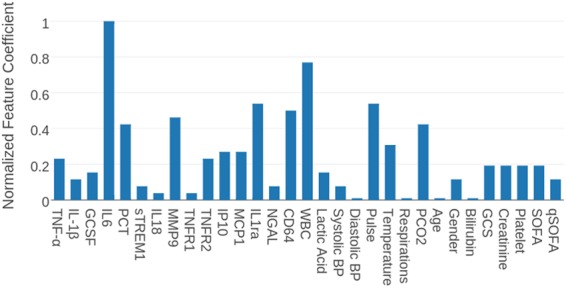
Normalized feature coefficients outputted by SVM for clinical adjudication label set. The absolute value of each feature coefficient in SVM corresponds to its relative importance.

We now focus on the results of our SVM model with feature selection in more detail since it achieved among the highest AUC relative to all the other models among all feature sets (specifically achieving an AUC of 0.807 when using feature selection on the entire feature set). To assess the relative importance of features in our model, we plot the coefficients outputted by SVM in Fig. 2. The top 11 features selected for the clinical adjudication label set in decreasing order of importance were IL-6, white blood cell count, IL-1ra, pulse, nCD64, MMP9, PCO2, PCT, temperature, and MCP1, IP10.

Because our goal is to predict whether or not a patient is in the early to peak phase of sepsis at the time of the biomarker measurement, an ideal model would take into account only EMR data up until the time of the biomarker measurement. However, we experimented with additionally including various subsets of the EMR data for times *after* the biomarker measurement. We specifically looked at subsets including 0, 1, 4, 8, 12, 16, 20, and 24 hours of EMR data collected after the time of the biomarker measurement.

**Figure 3:**
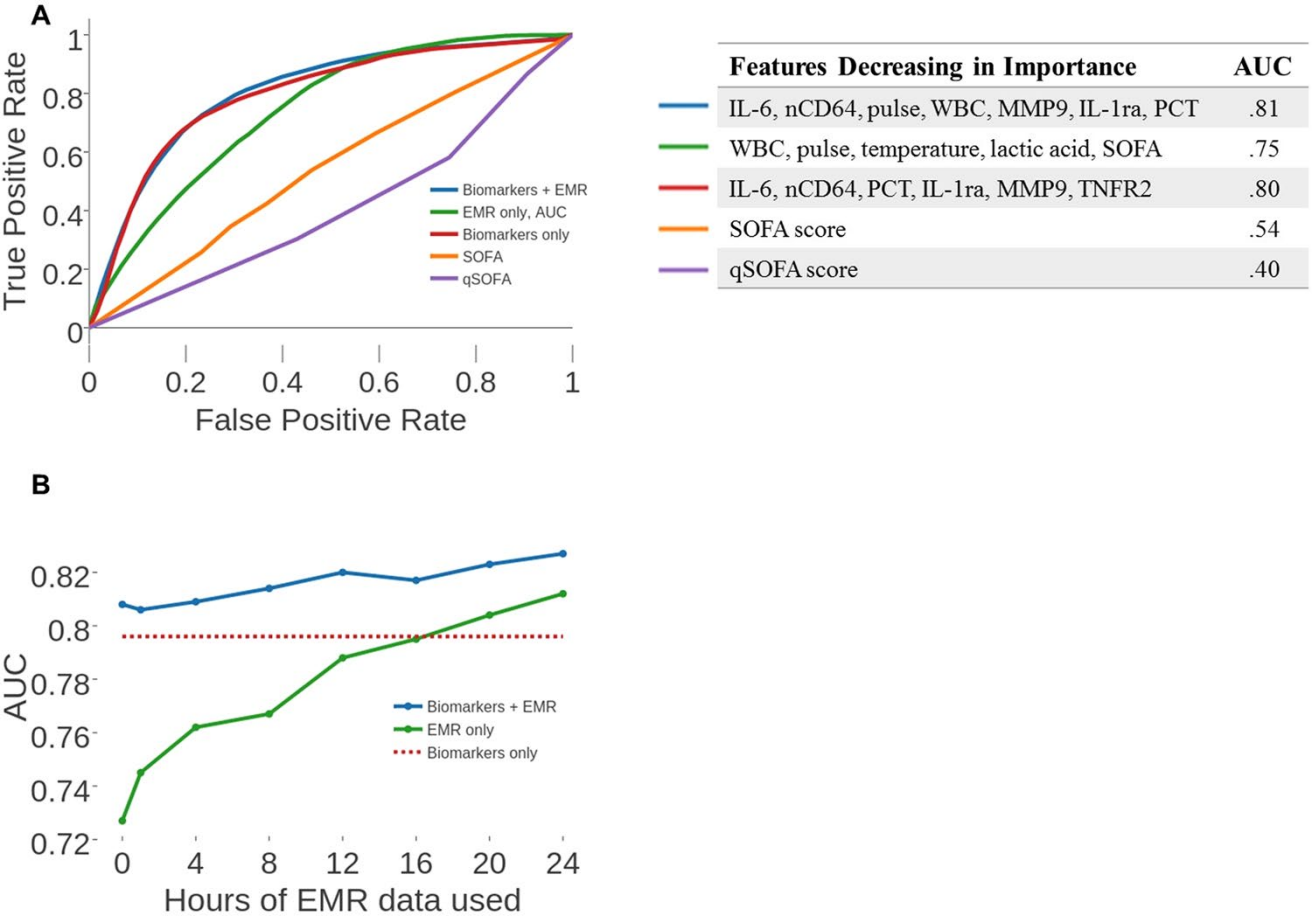
SVM w/feature selection performance as a function of time for clinical adjudication label set. (**A**) ROC curves are displayed for various feature sets. The EMR data that is used is constrained to up to 48 hours before the biomarker measurement and 1 hour after. **(B)** A plot of the AUC as a function of the number of hours of EMR data used post biomarker measurement.

ROC curves using 1 hour of EMR data post biomarker measurement are displayed in Fig. 3a. The curves in each figure are labelled according to their corresponding feature set. Additionally, ROC curves based on using SOFA and qSOFA scores as input features are displayed, as these are commonly-used metrics in the clinical evaluation of sepsis.

To better characterize the statistical difference of the improvement in the AUC obtained from the combination of biomarkers and EMR data versus only biomarkers or only EMR data, we calculated the proportion of our replications in which the combined model achieved a higher AUC than the other two models. We report these values in Table S4 and discuss how the calculations were performed in Materials and Methods.

To see how the AUC for each feature set varied as function of the number of hours after the sample measurement, for each time interval, we constructed a model incorporating the single sample biomarker measurements and EMR data collected up to either 0, 1, 4, 8, 12, 16, 20, and 24 hours after the sample measurement. This resulted in 8 distinct models for each set of features. After constructing a model for each time interval for each set of features, we calculated the respective AUC values and plotted this data in Fig. 3b.

### SOFA score

**Figure 4:**
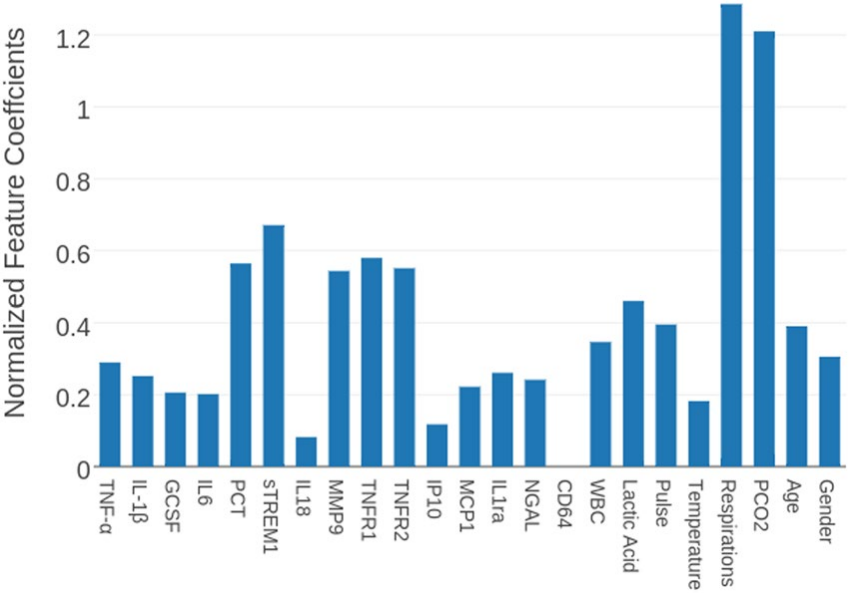
Normalized feature coefficients outputted by Random Forest for SOFA score label set.

In addition to predicting if a patient is in the early to peak phase of sepsis, we experimented in predicting the SOFA score^3^. We trained four regression models (support vector regression, logistic regression, random forest, and adaboost) that considered 23 features (which excluded any criteria used in generating the SOFA score) to predict the log of the SOFA score. We calculated the Spearman correlation between the predictions of the linear regression model and the SOFA score. We report the results for each algorithm with and without feature selection in Table 5. The feature rankings outputted by random forests (the best performing model) are displayed in Fig. 4 while the feature rankings outputted by the remaining methods are reported in Table S2.

**Table 5:** Regression method’s Spearman coefficient as a function of feature set. The Spearman coefficient is calculated for each regression technique with and without its respective feature selection method. The EMR data that is used is constrained to up to 48 hours before the biomarker measurement and 1 hour after.

Regression Model	All features (μ,σ)	EMR (μ,σ)	Biomarkers (μ,σ)
Logistic Regression	0.62, 0.10	0.38,0.13	0.52, 0.11
Logistic Regression w/ feature selection	0.60,0.10	0.36, 0.14	0.52, 0.11
SVM	0.63, 0.09	0.45, 0.12	0.53, 0.11
SVM w/ feature selection	0.59. 11	0.47, 0.11	0.46, 0.12
Random Forest	0.69, 0.08	0.50, 0.11	0.56, 0.10
Random Forest w/ feature selection	0.69,0.07	0.51, 0.11	0.57,0.10
Adaboost	0.67, 0.08	0.47, 0.12	0.57, 0.10
Adaboost w/ feature selection	0.62, 0.09	0.47, 0.12	0.56, 0.10

### Sepsis stratification

**Figure 5:**
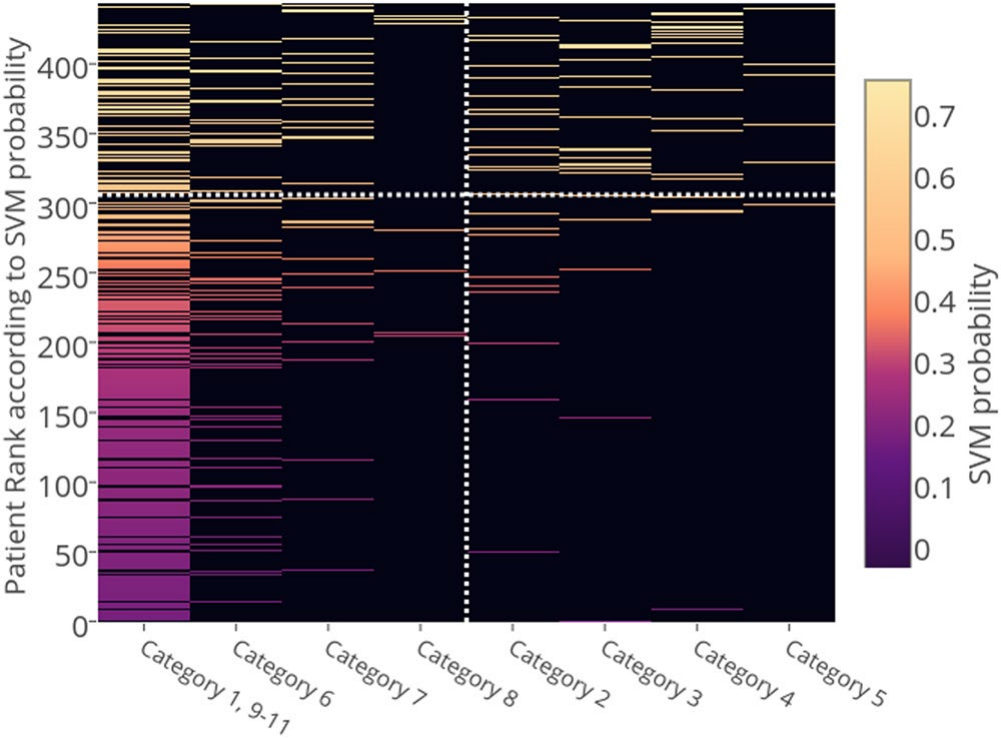
Heatmap for clinical adjudication label set. The x-axis corresponds to which category the patient was adjudicated to be in (see Materials and Methods) and the y-axis corresponds to the rank of the patient according to the probability outputted by SVM. For each patient, a line is plotted. The x coordinate of the line corresponds to which category the patient is labelled to be in and whose y coordinate corresponds to the rank of the patient is plotted. The color of this line is based on the probability that the patient is in early or peak phase according to SVM. The mapping from probability to color is displayed at the right of the figure. The vertical dotted white line separates the septic (categories 2–5) from the non-septic patients (categories 1, 6–11). The horizontal dotted line represents the patient whose probability of having sepsis was 0.50 according to SVM. The upper left quadrant represents the false positives, the upper right quadrant represents the true positives, the lower left quadrant represents the true negatives, and the lower right quadrant represents the false negatives. The black background corresponds to empty entries in the heatmap.

**Figure 6:**
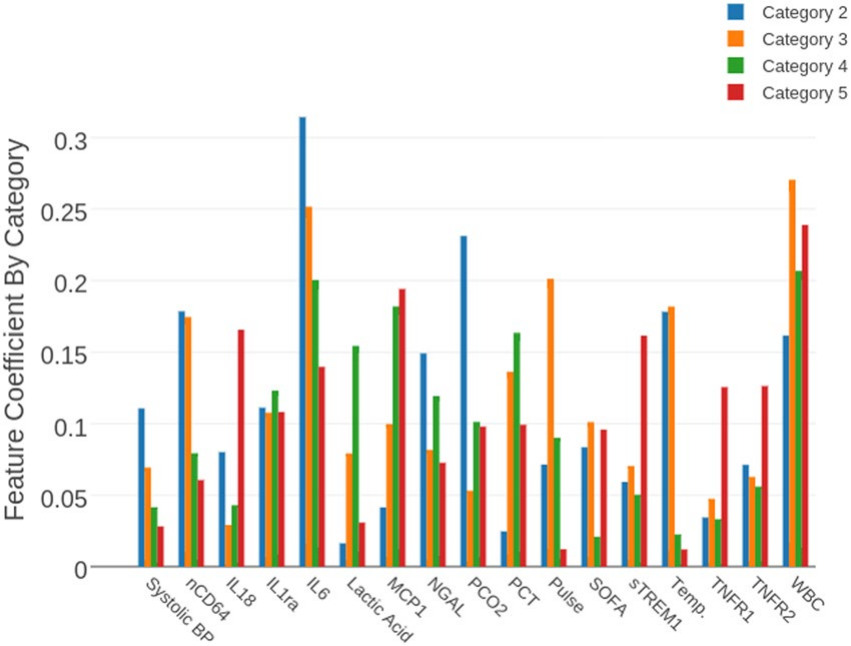
Feature coefficient outputted by SVM as a function of category.

We are interested in whether more precise stratification may serve as a mechanism for timelier recognition of sepsis as well as more tailored and timely intervention for each individual patient. To qualitatively examine the potential for our model to perform stratification in real-time, we generated a heatmap (Fig. 5). The x-axis corresponds to the labels assigned during the adjudication process and the y-axis displays the rank of each patient, which is determined according to the probability that they have sepsis generated by our SVM model. We summarize the ability of the model to predict sepsis within each label category in Table 6. We report sensitivity/specificity values for each category (sensitivity for categories 2–5–early to peak phase sepsis–and specificity for categories 1, 6–11–not septic or recovery phase sepsis). Each value is reported using 0.50 as a cutoff score for sepsis vs. not sepsis, with categories 4 and 5 achieving the highest values. The mean and standard deviation of the probability outputted by SVM is also presented in Table 6. To understand why our model performs better in some categories as opposed to others, we also constructed a bar chart plotting the weight of the most significant features as a function of category. (Fig. 6).

**Table 6:** SVM performance as a function of category. We report the sensitivity/specificity and average probability outputted by SVM for each category. Each number in the second column refers to sensitivity if the row corresponds categories 2–5 (patients considered positive) and specificity if the row corresponds to categories 1, 6–11 (patients considered negative).

Category	Sensitivity/Specificity	SVM probability (µ, σ)	Number of samples
1, 9–11	0.82	(0.32,0.16)	269
2	0.62	(0.52,0.15)	26
3	0.79	(0.55,0.14)	24
4	0.85	(0.62,.13)	20
5	1.0	(0.62,0.07)	6
6	0.67	(0.39,0.17)	58
7	0.42	(0.50,0.17)	58
8	0.57	(0.40,0.18)	7

## Discussion

Much has been presented in the literature describing the search for the ideal biomarker with adequate diagnostic value in the identification of sepsis; however, no such biomarker has yet been observed. We hypothesize that the ultimate tool for sepsis identification will require multiplexed biomarker sampling in addition to the traditionally-monitored parameters including vital signs, blood cell counts, and non-specific inflammatory markers. In this report, we demonstrate the contribution of a combination of non-traditional biomarker measurements to identify hospitalized adult patients in the early to peak phase of sepsis by a supervised machine learning model and examine the value this approach may provide in stratification of the sepsis syndrome, which may subsequently guide fundamental understanding of sepsis pathophysiology and/or serve as a new framework for targeted intervention.

We began by independently examining each of the 15 biomarkers selected for our study. Quantitative assessment of individual biomarkers (e.g. AUC) (Table 2) indicates a variable range in terms of their predictive power and signals an opportunity for improvement. Although some studies have reported high AUCs using individual biomarkers, these studies generally use homogenous patient populations to exclude for confounding variables. Since we did not omit any patients from our analysis, it is unsurprising that none of the individual biomarkers provided high discriminative power.

Similar analysis reinforces that the traditional parameters which serve to inform the clinician (e.g. leukocyte count, lactic acid, and vital signs) are similarly non-informative as independent measures (Table 3). Indeed, this observation is consistent with the notion that there is processing of information, (potentially partially subconscious) in the mind of the experienced clinician, which synthesizes several of these measures to assess the overall clinical picture and to arrive at a conclusion regarding the presence of sepsis. It is the very spirit of our hypothesis that novel biomarker measurements should be weighed in combination; however, as the volume of data input increases, the method for processing this data must become more sophisticated. This is the very inspiration for our application of machine learning to the sepsis problem.

To test our hypothesis that the ultimate tool for identifying patients in the early to peak phase sepsis would require a combination of biomarkers and traditionally-monitored EMR parameters, we applied various machine learning algorithms to various subsets of our data. First we observe (Table 4) that among all algorithms with and without feature selection, models which employed solely non-traditional biomarkers always outperformed models which used solely EMR data. This is an exciting observation, as it hints to the potential value of the information contained solely within the biomarkers. Meanwhile, models combining the biomarkers and EMR data with and without feature selection always outperformed models with either of the limited feature sets.

Table S3 depicts the statistical difference between our final model that was chosen (SVM with feature selection amongst all features) and all other models. While Adaboost slightly outperforms SVM, we chose SVM because it is less computationally expensive to train and more interpretable than Adaboost.

To appreciate the diagnostic power of a machine learning approach, we compare the ROC curves from our final model amongst various feature sets utilizing one hour of EMR data after the time of the biomarker measurement. We again observe that using non-traditional biomarkers in conjunction with EMR data outperforms all other feature sets (Fig. 3a). Furthermore, the AUC values for the model using biomarkers and EMR data values were greater than the AUC values for the model using solely EMR data in 78% of our cross validation replications, indicating the predictive power of the biomarkers.

As expected with using future data to predict a prior state, there is a slight increase in the AUC as larger time windows of data from the EMR are considered (Fig. 3b). As expected, we see improvement in the performance (i.e. increasing AUC) of any algorithm which includes EMR data as the time window for sampling data from the EMR is increased. It is also important to note that the AUC when using only non-traditional biomarker features (specifically IL-6, nCD64, IL-1ra, PCT, MCP1, and G-CSF – the biomarkers chosen by SVM’s feature selection process) was equal to the AUC when using solely EMR data only when 16 hours of EMR data following the time of sample acquisition was utilized (Fig. 3b).

Overall, these observations suggest that 1) using biomarkers in conjunction with EMR data can more accurately predict if a patient is in the early to peak phase of sepsis as opposed to using solely EMR data and 2) biomarker analysis with machine learning may provide insight into the patient’s condition more quickly than current traditional clinical data, provided that assays for these biomarkers could be performed rapidly.

Furthermore, the comparison of four feature selection methods reinforces the conclusion that non-traditional biomarkers are a more valuable source of data for identifying patients in the early to peak phase of sepsis. All methods ranked a modest proportion of the non-traditional biomarkers similarly or higher than the EMR features amongst all label sets (Fig. 2, Tables S1).

Our results in predicting the SOFA score, an indicator of organ dysfunction, by regression analysis (Table 5) further cement the theme of the predictive power of the biomarkers. We notice that for all regression techniques, the Spearman correlation is always highest when using biomarkers in conjunction with EMR data, followed by using only biomarkers, followed by using only the EMR data. We again notice that the four feature selection methods rank a modest proportion of biomarkers similarly or higher than the EMR features (Fig. 4 and Table S2). The fact that the biomarkers still show predictive power for a relatively distinct label set is a promising indicator of their significance. It is also interesting to note that the biomarkers ranked highly (PCT, sTREM1, TNFR1, TNFR2, MMP9) are different from the biomarkers chosen when predicting on the clinical adjudication label set. This result is intuitive, since it is expected that different markers would correlate to organ dysfunction than those with high correlation to the onset of sepsis.

The notion that stratification of the septic patient may provide insight into disease process and create opportunities for more directed intervention has been a topic of discussion in the literature. Our adjudication processes were directed at exploring this notion by stratifying subjects into categories that describe the state of their illness in more detail than “sepsis” vs. “no sepsis.” Our hypothesis that more sophisticated (i.e. machine learning) analysis of non-traditional biomarkers and clinical measures may support this approach is examined in the heatmap in Fig. 5.

As we examine the heatmap, we first notice that our model is more confident in predicting the more severe stages of sepsis. Specifically, the model predicts severe sepsis (category 4) with a sensitivity of 0.83 (n = 20), septic shock (category 5) with a sensitivity of 100% (n = 6), and sepsis (category 3) with a sensitivity of 79% (n = 24). We also notice that our model is less confident in identifying patients who are in the recovery phrase of their corresponding septic state as not being in the early to peak phase of sepsis. The lower specificity for patients in categories 6, 7, and 8 (patients who are recovering from sepsis, severe sepsis, and septic shock, respectively) reflects the difficulty in classifying subjects as within or recovering from the most severe phase of their illness as some parameters used to make this classification may be improving while other parameters may be deteriorating. This is supported by the findings of Rhee *et al*.^33^, who conducted a large scale sepsis diagnosis study and concluded that inter-observer agreement among physicians in diagnosing sepsis and its respective stage is poor. We also observe that within categories 6, 7, and 8 our model is more likely to predict sepsis in patients who are recovering from more severe forms of sepsis. Specifically, the model predicts subjects recovering from *severe* sepsis (category 7) to be septic with a probability of 50%, subjects recovering from septic shock (category 8) with a probability of 50%, and subjects recovering from sepsis (category 6) with a probability of 37%. This is consistent with both ours and the literature’s expectation^34^ that the biomarkers reflecting systemic inflammation are more likely to remain abnormal in patients recovering from severe sepsis and septic shock as opposed to sepsis.

The heatmap also identifies areas of weakness in our model, such as the false positives (subjects who are not labeled as septic in the adjudication process but considered septic by the model) in the upper left quadrant and false negatives (subjects who were labeled as septic but not identified as septic by the model) in the lower right quadrant. Both false positives and false negatives may be attributed to confounding factors such as the presence of comorbidities, including systemic inflammatory conditions other than sepsis which may influence levels of inflammatory mediators without progressing to full blown sepsis. Table 1 summarizes the presence of these potential confounding factors in our population. For example, 42% of our population not in the early to peak phase of sepsis had an infection and satisfied at least two out of four SIRS criteria (thus meeting the classic definition of sepsis), and a non-negligible portion of the non-septic population had various chronic conditions. 17% had cancer, 19% had diabetes, 9% had chronic kidney disease, and 10% had COPD. These statistics are an artifact of treating patients in the recovery phase of sepsis as negative examples. However, by treating patients in the recovery phase of sepsis as negative examples, we built a model that discriminates patients in the early to peak phase of sepsis from both non-septic patients and patients in the recovery phase of sepsis. Understanding how these confounding factors affect the biomarkers and other EMR data parameters is an important topic of research for future work.

Meanwhile, our analysis suggests that a “one size fits all” model may not be able to accurately predict sepsis. Fig. 6 summarizes feature importance as evaluated by the model. It is clear that different biomarkers are considered more significant in the various stages of sepsis. For example, one notices that nCD64 is elevated in the earlier stages of the disease while MCP1 and PCT are elevated in the latter stages of the disease. We believe this observation supports the hypothesis that more sophisticated tools with greater volumes of data will be necessary in conjunction with a more descriptive stratification of the septic patient depending on their condition at the time of biomarker measurement. A larger-scale analysis which incorporates multiple measurements of multiple biomarkers may enable us to understand which biomarkers define the clinically-relevant phases of sepsis.

Finally, our model was not tested on an independently collected validation test set (but rather through randomly sampled folds via repeated 10-fold cross validation) and should be an area of future work. Furthermore, our model does not consider the nature nor appropriateness of the treatment administered to the subjects in our study. While this is a very complicated issue, further work may require complex analysis of the administration of antibiotics, intravenous fluids, vasopressors, and other relevant interventions as features in identification of patients at various stages of sepsis.

The analysis presented in this manuscript supports our hypothesis that the best tool for identifying patients in the early to peak phase of sepsis would require a combination of biomarkers and traditionally-monitored clinical parameters. Furthermore, the fact that significantly more EMR data is needed to achieve an AUC equivalent to that of a single measurement of multiple biomarkers is a promising indicator of the predictive power of biomarkers and their potential role in timely intervention. Finally, our methodology and subsequent analysis of stratifying patients based on their condition at the time of assessment with respect to the overall disease trajectory provided scientific insights that will enable improvement and ultimately convergence to a more precise, clinically-relevant, biomarker-based description of the course of the sepsis disease process.

## Materials and Methods

### Patient selection and data collection

All methods were performed in accordance with the relevant guidelines and regulations established by the Institutional Review Boards. Blood samples were acquired following protocols approved by the IRB of the University of Illinois at Urbana-Champaign and Carle Foundation Hospital (CFH) in Urbana, IL. A waiver of consent was obtained from the IRBs. Blood samples were randomly collected from patients with a physician-initiated blood culture order. Our patient population consists of patients from emergency department, medical care, oncology, surgical care, cardiac care, and intensive care. Venous blood collected for routine hematology analysis by a hospital phlebotomist in a 4 mL EDTA-coated vacuum tube was transferred to a clean 1.5 mL vial from the remaining blood in the original collection vial following routine processing by an automated hematology analyzer. These transferred samples were de-identified by a research coordinator and assigned a unique sample identification code. No restrictions were placed on the timing of sample collection relative to hospital admission, and no samples which fit the criteria (drawn in conjunction with venous blood collection for bacterial culture) were excluded from this study for any reason. We consciously did not restrict the timing of the sample collection to make the model as generalizable as possible. We believe that even if we did restrict the timing of the sample to a specific time window, it would not standardize the phase of the disease each patient is in. A histogram of the time of the sample measurement is displayed in Figure S1 for reference.

Data that are not considered protected health information for subjects who had been discharged or deceased were retrieved from the electronic medical record (EMR) by a data engineer and reported to the research team with sample identification code corresponding to each blood sample.

### Biomarker measurement process

Plasma protein biomarkers were measured using the Magnetic Luminex Assay technology, which is a bead-based multiplex assay. Within 24 hours of receiving the blood samples, portions were chilled (4 °C) and centrifuged for 5 minutes at 1 RCF to remove all blood cells. The resulting plasma was collected and frozen (−80 °C) for later analysis. Prior to performing each assay, plasma samples were thawed and allowed to warm to ambient temperature. For each assay, 40 plasma samples and 8 standard samples were prepared in duplicate and measured. Neutrophil CD64 was measured using a standard Leuko64 assay from Trillium Dx. Mean coefficient of variation values for each biomarker across all measurements are reported in Table S5.

### Adjudication process

In order to provide an accurate “gold standard” label for each subject analyzed, it was necessary to define their clinical condition at the time the corresponding blood sample was collected. Furthermore, as described previously, we sought to classify patients with more specific descriptions than “sepsis” vs. “no sepsis.” This includes the temporal relationship of the blood sample to the point in hospitalization where the patient could be reasonably considered septic as well as when they were in the most severe condition experienced during the course of their hospitalization. The clinical adjudication process, therefore, sought to assign each subject to one of the following eleven categories:

1. Never septic
2. Approaching sepsis, severe sepsis, or septic shock with evidence of deterioration
3. Currently experiencing sepsis, where sepsis refers to sepsis events that are not severe sepsis or septic shock
4. Currently experiencing severe sepsis
5. Currently experiencing septic shock
6. Recovering from sepsis with some evidence of systemic inflammation
7. Recovering from severe sepsis with some evidence of systemic inflammation
8. Recovering from septic shock with some evidence of systemic inflammation
9. Recovered from sepsis without evidence of systemic inflammation
10. Recovered from severe sepsis without evidence of systemic inflammation
11. Recovered from septic shock without evidence of systemic inflammation

A visual summarization of the categories is displayed in Figure S2. The peak corresponds to the apex of disease severity, which is determined according to clinical judgement. The intention of the categories is twofold. First, it enables us to capture if a patient is in the early to peak phase of their disease (categories 2–5) or not (categories 1, 6–11). Second, it enables us to characterize the performance and characteristics of our models as a function of all individual categories.

Our categories are similar to those defined by Otto *et al*.^35^, who attempted to characterize sepsis into a set of four phases. Each phase was defined by the days prior to diagnosis or during sepsis, which in turn was defined by mortality peaks that were separated by two distinct nadirs. Phase 0 (1 to 10 days prior to diagnosis) and phase 1 (1 to 5 days after diagnosis) were characterized by either higher SOFA scores, PCT values, or mortality rates than phase 2 (6 to 15 days after diagnosis) and phase 3 (16 to 150 days after diagnosis). This study sets a precedent for our categorization of septic patients as either in the early, peak, or recovery phase, of the disease.

### Data processing

#### Vitals

Since each vital sign was measured over time for each patient’s entire hospital stay and our analysis only concerns the information near the time point of the biomarker measurement, we wanted to conglomerate this data into a single variable. To do this, we took the Riemann sum between the particular vital function and its corresponding threshold value normalized over time. This Riemann sum was constrained to a domain where the vital function’s values were abnormal, and to account for differences in the ranges of measurements, the height of each rectangle in the Riemann sum was transformed to a percent difference. We also experimented with various time windows. Since we transformed our label set to be relevant around the time of the biomarker measurement, each time window is centered at the time of the biomarker measurement. We used vital data up to 48 hours taken before the biomarker measurement (if available) and experimented with windows of 0,1,4, 8, 12, 16, 20, and 24 hours after the biomarker measurement. A mathematical formulation for this feature transformation is detailed in Supplementary Information.

#### Biomarkers

Plasma samples were assayed in 40 sample batches and normalized to standard values using batch-specific calibration curves. To ensure robustness to outliers, we converted the biomarker concentration values to quantiles by performing quantile normalization. If certain biomarkers were calculated to be less than or greater than a value due to being below or above its lower or upper limit of detection, we calculated the conditional expectation, *E[X | X* > *x]* or *E[X | X* < *x]* of its concentration.

### Feature selection

For logistic regression and naive Bayes, we performed LASSO penalized logistic regression^36^ on our dataset and used the features with nonzero coefficients. For support vector machines, random forests, and adaboost, we ranked each of the features according to a certain metric. For SVM, we ranked features according to their weight vector coefficients; for random forests and for adaboost, we ranked the features according to their mean decrease in predictive accuracy. We then added each feature with the largest coefficient to our current feature set, removed that feature from the previous feature set, calculated the AUC of each model with its current feature set, and repeated this process until we reached the maximum AUC value for each model.

### Algorithm training

All algorithms were run treating patients in categories 2–5 as positive examples and patients in categories 1, 6–11 as negative examples. This executes the intention of classifying a patient as either in the early to peak phase of sepsis/severe sepsis/septic shock or either recovering from sepsis/severe sepsis/septic shock or not septic. Algorithms were trained using 10-fold cross-validation. For a given set of features, we repeated this entire procedure 1000 times in order to generate different cross-validation folds. This provides a measure of the statistical uncertainty of our AUC estimates. We then calculated the average cross-validation AUC estimates for each algorithm and each set of features with and without feature selection.

### Model comparison

To compare the predictive power of two models, we report the percentage of differences in paired AUC values that are above 0 and the mean difference between all paired AUC values. More specifically, 9/10ths of the data was randomly sampled, 2 models were trained using this subset of the data, and then each model was evaluated in terms of AUC on the remaining 1/10th of the data. We repeat this process 1000 times yielding 10,000 separate AUC values (10 AUC values are calculated per iteration across 1000 total iterations). Note that each AUC for each model corresponds to the same fold of data, so the 10,000 separate AUC values for each model are paired. To compare the ROC curves between two models, we now take the element wise difference between corresponding AUC values for each model and calculate the percentage of differences that are above 0 and the mean value of this vector. We report the percentage of paired AUC values above 0 and mean difference in paired AUC values in Tables S3 and S4. Table S3 reports the percentage of paired AUC values above 0 and mean difference in paired AUC values between SVM with feature selection and all other models. This provides a comparison of the statistical difference between our final model that was chosen (SVM with feature selection amongst all features) and all other models. Table S4 reports the percentage of paired AUC values above 0 and mean difference in paired AUC values between 3 SVM models (one using biomarker and EMR data, one using only biomarker data, and one using only EMR data) to provide a comparison of the statistical difference between using different sets of features.

## Electronic supplementary material

Supplementary Information
