# Publisher Correction: Combining Biomarkers with EMR Data to Identify Patients in Different Phases of Sepsis

**DOI:** 10.1038/s41598-019-53691-4

**Published:** 2019-11-19

**Authors:** Ishan Taneja, Bobby Reddy, Gregory Damhorst, Sihai Dave Zhao, Umer Hassan, Zachary Price, Tor Jensen, Tanmay Ghonge, Manish Patel, Samuel Wachspress, Jackson Winter, Michael Rappleye, Gillian Smith, Ryan Healey, Muhammad Ajmal, Muhammad Khan, Jay Patel, Harsh Rawal, Raiya Sarwar, Sumeet Soni, Syed Anwaruddin, Benjamin Davis, James Kumar, Karen White, Rashid Bashir, Ruoqing Zhu

**Affiliations:** 1 Department of Bioengineering, University of Illinois at Urbana Champaign, 1270 Digital Computer Laboratory, 1304W, Springfield Ave., 61801 Urbana, IL USA; 2 Department of Statistics, University of Illinois at Urbana Champaign, Illini Hall, 725S Wright St #101, 61820 Champaign, IL USA; 3 Biomedical Research Center, Carle Foundation Hospital, 61801 Urbana, IL USA; 4 Prenosis Inc., 210 Hazelwood Drive, Suite 103, 61822 Champaign, IL USA

Correction to: *Scientific Reports* 10.1038/s41598-017-09766-1, published online 07 September 2017

The Competing Interests statement in the original version of this Article incorrectly stated that “The authors declare that they have no competing interests”. The Competing Interests statement now reads:

“Ishan Taneja, Bobby Reddy Jr. and Samuel Waschpress work for Prenosis Inc.. Jackson Winter worked at Prenosis at the time of submission but has since left and is now a graduate student at the University of Illinois at Urbana-Champaign. All other authors report no competing interests.”

In addition, the author Jackson Winter was incorrectly given as Jake Winter.

These errors have now been corrected in the HTML and PDF versions of the Article, and in the accompanying supplementary material.

