# Supplementary material for: Combining Biomarkers with EMR Data to Identify Patients in Different Phases of Sepsis
Source: Sci Rep. 2017 Sep 7;7:10800. doi: 10.1038/s41598-017-09766-1 (PMC5589821; doi:10.1038/s41598-017-09766-1)
Supplement: Supplementary file 1 — Supplementary Information [file 41598_2017_9766_MOESM1_ESM.docx]

**Combining Biomarkers with EMR Data to Identify Patients in Different Phases of Sepsis**

**Authors:** Ishan Taneja^1,3,4^, Bobby Reddy Jr. ^1,3,4^, Gregory Damhorst^1,3^, Dave Zhao^2^, Umer Hassan^1,3^, Zachary Price^1,3^, Tor Jensen^1,3^, Tanmay Ghonge^1,3^, Manish Patel^1,3^, Samuel Wachspress^1,3,4^, Jackson Winter^1,3,4^, Michael Rappleye^1,3^, Gillian Smith^1,3^, Ryan Healey^1,3^, Muhammad Ajmal^3^, Muhammad Khan^3^, Jay Patel^3^, Harsh Rawal^3^, Raiya Sarwar^3^, Sumeet Soni^3^, Syed Anwaruddin^3,^ Benjamin Davis^3^, James Kumar^3^, Karen White^3^, Rashid Bashir^1,3*^, Ruoqing Zhu^2*^

**Supplementary Information**

*Figures*

**
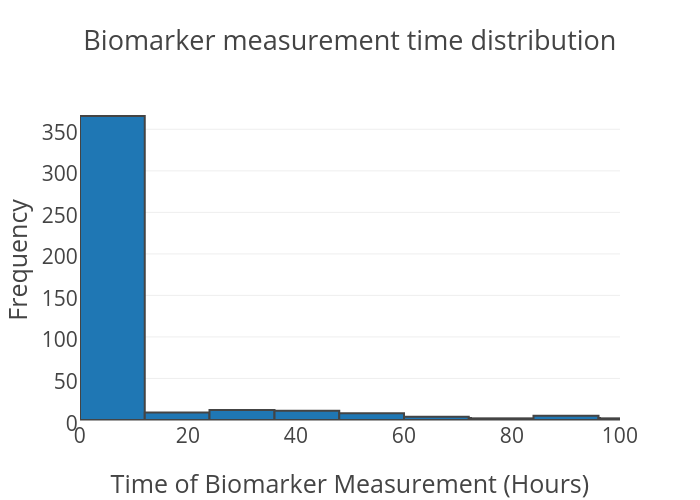
**

**Figure S1. Biomarker measurement time distribution.** Majority of sample measurements (80%) are within the first 6 hours of admission.


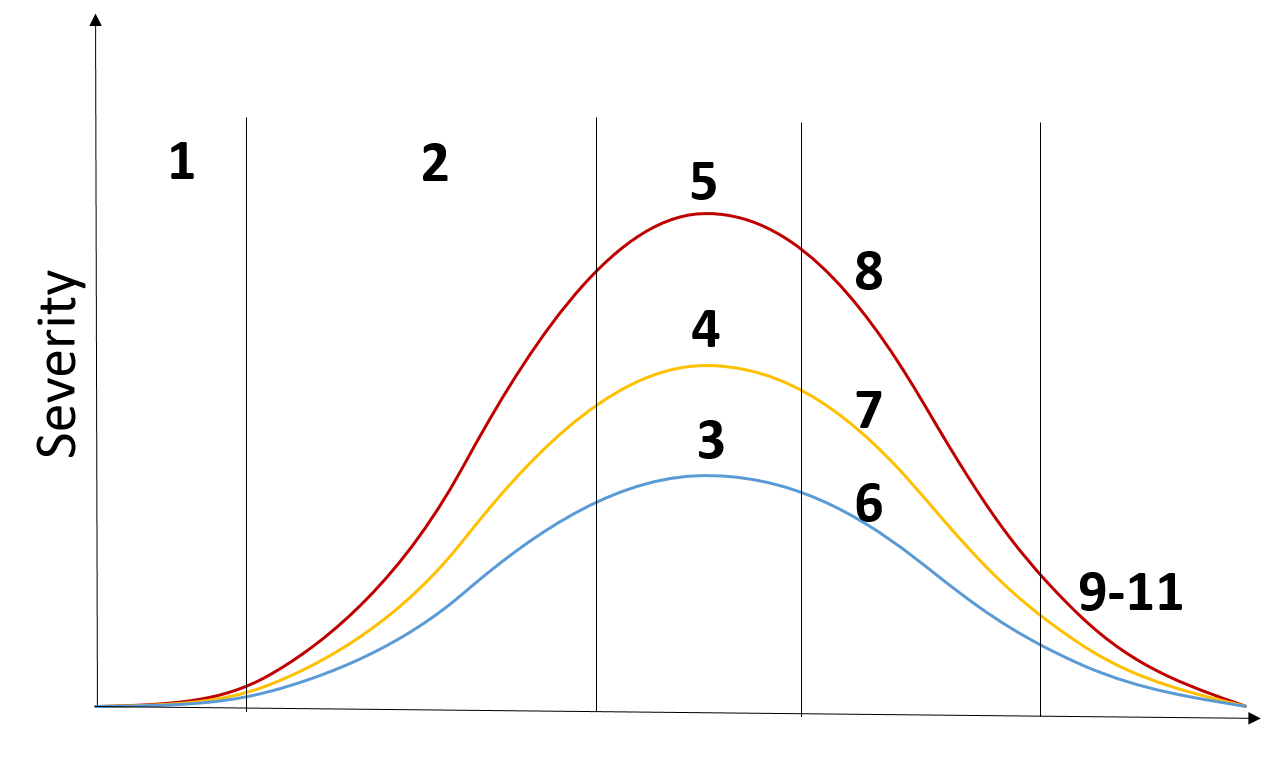


**Figure S2. Patient disease state categorization as a function of sepsis severity.** The blue/orange/red curve represents a hypothetical patient’s severity of sepsis/severe sepsis/septic shock respectively as a function of time, where the apex of the curve corresponds to their worst case state. The vertical lines partition the course of the disease into various categories. 1 corresponds to not septic, 2 corresponds to approaching the peak state of sepsis/severe sepsis/septic shock, 3-5 corresponds to within the peak state of sepsis/severe sepsis/septic shock respectively, 6-8 corresponds to recovering from sepsis/severe sepsis/septic shock respectively, and 9-11 corresponds to significantly recovering from sepsis/severe sepsis/septic shock respectively.

*Tables*

| Feature | LASSO coefficient | SVM coefficients | Random Forest | Adaboost |
| --- | --- | --- | --- | --- |
| TNF-α | 0 | -.06 | -1.31 | .55 |
| IL-1β | 0 | -.03 | .38 | .29 |
| GCSF | 0 | -.04 | -.28 | 1.04 |
| IL-6 | 1.93 | -.26 | 6.81 | 38.07 |
| PCT | 0 | -.11 | -2.86 | 5.96 |
| sTREM1 | 0 | .02 | -2.15 | 1.15 |
| IL18 | 0 | -.01 | -.11 | .73 |
| MMP9 | 0 | -.12 | -2.33 | 1.49 |
| TNFR1 | 0 | -.01 | -3.16 | 1.11 |
| TNFR2 | 0 | -.06 | -4.96 | 3.15 |
| IP10 | 0 | -.07 | -1.98 | 1.56 |
| MCP1 | 0 | -.07 | 1.48 | 2.90 |
| IL-1ra | 0.19 | -.14 | 2.94 | 12.37 |
| NGAL | 0 | -.02 | 1.10 | 2.35 |
| CD64 | 0.37 | -.13 | .98 | 4.25 |
| WBC | 0.22 | -.20 | 1.11 | 4.05 |
| Lactic Acid | 0 | -.04 | -.04 | .15 |
| Systolic Blood Pressure | 0 | .02 | -1.84 | .017 |
| Diastolic Blood Pressure | 0 | .00 | -.96 | .74 |
| Pulse | 0.25 | -.14 | 2.17 | 9.40 |
| Temperature | 0 | -.08 | 3.04 | 3.50 |
| Respirations | 0 | .00 | -.04 | 1.91 |
| PCO_2_ | 0 | .11 | 2.78 | 2.51 |
| Age | 0 | .00 | 2.11 | 1.11 |
| Gender | 0 | .03 | -1.71 | .12 |
| Bilirubin | 0 | .00 | -2.30 | .15 |
| Glasgow Coma Scale | 0 | .05 | .33 | .26 |
| Creatinine | 0 | .05 | -1.00 | .15 |
| Platelet | 0 | -.05 | -1.09 | .29 |
| SOFA score | 0 | -.05 | 1.92 | 1.06 |
| qSOFA score | 0 | -.03 | 2.44 | 1.55 |

**Table S1. Feature coefficients/importance for clinical adjudication label set**

| Feature | LASSO coefficient | SVM coefficient | Random Forest | Adaboost |
| --- | --- | --- | --- | --- |
| TNF-α | 0 | .03 | .05 | .42 |
| IL-1β | 0 | -.05 | -.49 | .57 |
| GCSF | 0 | .04 | -1.15 | .75 |
| IL-6 | 0 | .10 | -1.21 | .37 |
| PCT | 0.65 | .14 | 3.99 | 15.44 |
| sTREM1 | 0.10 | .21 | 5.52 | 4.53 |
| IL18 | 0 | -.03 | -2.93 | .20 |
| MMP9 | -0.14 | -.17 | 3.69 | 6.62 |
| TNFR1 | 0.76 | .19 | 4.21 | 13.94 |
| TNFR2 | 0 | .13 | 3.80 | 1.40 |
| IP10 | 0 | .02 | -2.42 | 3.24 |
| MCP1 | 0 | .06 | -.92 | 6.80 |
| IL-1ra | 0 | -.09 | -.36 | .55 |
| NGAL | 0 | .01 | -.64 | .10 |
| CD64 | 0.41 | -.01 | -4.11 | 8.16 |
| WBC | 0.23 | -.01 | .87 | 1.11 |
| Lactic Acid | 0.28 | .13 | 2.49 | 12.92 |
| Pulse | 0 | -.07 | 1.57 | .11 |
| Temperature | 0 | .03 | -1.49 | .03 |
| Respirations | 0.26 | .32 | 14.36 | 9.35 |
| PCO_2_ | -1.57 | -.19 | 13.27 | 8.65 |
| Age | 0 | .01 | 1.50 | 5.53 |
| Gender | 0.007 | .02 | .28 | .007 |

**Table S2. Feature coefficients/importance for SOFA label set**

| Algorithm | All features | EMR | Biomarkers |
| --- | --- | --- | --- |
| **Logistic Regression** | (.75,.03) | (.83,.09) | (.74,.03) |
| **Logistic Regression w/ feature selection** | (.70,.02) | (.83,.08) | (.66,.02) |
| **SVM** | (.67,.02) | (.78,.08) | (.73,.02) |
| **SVM w/ feature selection** | NA | (.78,.06) | (.60,.01) |
| **Random Forest** | (.71,.03) | (.81,10) | (.83,.06) |
| **Random Forest w/ feature selection** | (.69,.03) | (.78,.09) | (.75,.04) |
| **Adaboost** | (.47,-.001) | (.73,.06) | (.64,.02) |
| **Adaboost w/ feature selection** | (.45,-.002) | (.69,.05) | (.56,.01) |
| **Naive Bayes** | (.80,.04) | (.87,.11) | (.77,.04) |
| **Naive Bayes w/feature selection** | (.62,.01) | (.79,.08) | (.65,.01) |

**Table S3. Percentage of paired AUC differences above 0 and mean value of paired AUC differences between our final chosen model (SVM w/ feature selection amongst all features) and all other combinations of algorithms and feature sets.**

| Feature Set Comparison | Percentage of paired AUC difference values above 0 |  |  |  |
| --- | --- | --- | --- | --- |
| Biomarker + EMR vs. EMR only | (.78,.06) |  |  |  |
| Biomarker + EMR vs. Biomarker only | (.60,.01) |  |  |  |
| Biomarker only vs. EMR only | (.72,.05) |  |  |  |

**Table S4. Percentage of paired AUC differences above 0 and mean value of paired AUC differences between specific pairs of feature sets.**

| Biomarker | Mean CV (%) |  |  |  |
| --- | --- | --- | --- | --- |
| TNF-α | 5.52 |  |  |  |
| IL-1β | 4.72 |  |  |  |
| GCSF | 8.87 |  |  |  |
| IL-6 | 5.16 |  |  |  |
| PCT | 5.46 |  |  |  |
| sTREM1 | 4.97 |  |  |  |
| IL18 | 4.68 |  |  |  |
| MMP9 | 4.96 |  |  |  |
| TNFR1 | 5.36 |  |  |  |
| TNFR2 | 6.40 |  |  |  |
| IP10 | 6.70 |  |  |  |
| MCP1 | 5.17 |  |  |  |
| IL-1ra | 7.37 |  |  |  |
| NGAL | 5.18 |  |  |  |
| CD64 | 5.37 |  |  |  |

**Table S5. Mean CV for each biomarker across all measurements.**

*Methods*

Lasso:

LASSO fits a generalized linear model with a L1 norm regularization term to augment the sparsity of coefficients. Specifically, the following penalized logistic regression problem is being solved:

$$\min_{\left( \beta_{0},\beta\right)\in R^{p+1}}-\left[ \frac{1}{N}\sum_{i=1}^{N} y_{i}\cdot\left( \beta_{0}+x_{i}^{T}\beta\right)-\log\left( 1+e^{\left( \beta_{0}+x_{i}^{T}\beta\right)} \right) \right]+\lambda||{\beta||}_{1}.$$

Feature transformation for vitals:

Let *x* be the time of the biomarker measurement. Let *a* represent the time window used before the biomarker measurement and let *b* represent the time window used after the biomarker measurement. Let *j* represent the particular vital (i.e pulse). Let *T_j_* be the set of discrete measurement times for vital *j* between [*x-a*, *x+b*], specifically restricted to times where the particular value of the measurement was considered abnormal. Let $f_{j}\left( t \right)$ represent the value of vital *j* at time *t*. Let Δ*t* represent the time difference between the measurement at time *t* and the next corresponding measurement for vital *j* (note that the next corresponding measurement does not necessarily need to be in *T_j_*). Let $g({j,f}_{j}\left( t \right))$ be a function outputting the particular threshold value for a given vital *j* and its corresponding value $f_{j}\left( t \right)$. For example, white blood cell count (WBC) measurements are considered abnormal if they are greater than 12E9/L or less than 4E9/L, so *g*(2E9,WBC) = 4E9 and *g*(16E9, WBC) = 12E9. So, for a given vital for a particular patient, their particular feature can be calculated as follows:

$$\frac{\sum_{t \in T_{j}} \frac{{|f}_{j}\left( t \right)-g({j,f}_{j}\left( t \right))|}{g({j,f}_{j}\left( t \right))}*\Delta t}{b-a}$$
